# Anti-CD2 Antibody-Coated Nanoparticles Containing IL-2 Induce NK Cells That Protect Lupus Mice *via* a TGF-β-Dependent Mechanism

**DOI:** 10.3389/fimmu.2020.583338

**Published:** 2020-12-16

**Authors:** David A. Horwitz, Aijing Liu, Sean Bickerton, Giuseppe Castaldo, Giuseppe Matarese, Tarek M. Fahmy, Antonio La Cava

**Affiliations:** ^1^ Keck School of Medicine, University of Southern California, Los Angeles, CA, United States; ^2^ General Nanotherapeutics, LLC, Santa Monica, CA, United States; ^3^ Department of Medicine, University of California Los Angeles, Los Angeles, CA, United States; ^4^ Department of Biomedical Engineering, Yale University, New Haven, CT, United States; ^5^ Dipartimento di Medicina Molecolare e Biotecnologie Mediche, Federico II University of Naples, Naples, Italy; ^6^ Istituto di Endocrinologia e Oncologia Sperimentale, Consiglio Nazionale delle Ricerche, Naples, Italy; ^7^ Department of Immunobiology, Yale University, New Haven, CT, United States

**Keywords:** autoimmunity, systemic lupus erythematosus, NK cells, immune regulation, nanoparticles, immunotherapy, cytokines

## Abstract

We recently reported that the treatment with nanoparticles (NPs) loaded with tolerogenic cytokines suppressed the manifestations of lupus-like disease induced by the transfer of donor CD4^+^ T cells from DBA/2 mice into (C57BL/6 × DBA/2)F_1_ (BDF1) mice. Although the protective effects were ascribed to the induction of adaptive CD4^+^ and CD8^+^ T regulatory cells, the results suggested that another population of immune cells could be involved. Here we report that NK cells critically contribute to the protection from lupus-like disease conferred by NPs to BDF1 mice, and that this effect is TGF-β-dependent.

## Introduction

Systemic lupus erythematosus (SLE) is a chronic, systemic autoimmune disease characterized by dysregulated immune responses that impair the mechanisms of peripheral immune tolerance that normally keep self-reactive immune cells under control (1). A significant contribution to the unbalanced immune homeostasis in SLE derives from a dysregulated production of cytokines, e.g. insufficient amounts of circulating anti-inflammatory cytokines and a preponderance of pro-inflammatory cytokines. In this context, interleukin‐2 (IL‐2) and transforming growth factor β (TGF-β) are deficient in SLE, and these cytokines play critical roles in immunoregulatory responses such as the induction and maintenance of CD4^+^ and CD8^+^ T regulatory cells (Tregs) (2–5).

Our group and others have reported that the conditioning of immune cells with IL‐2 and TGF-β could represent a therapeutic approach for the restoration of unbalanced immune homeostasis in SLE [reviewed in (6–9)]. More recently, we showed that the delivery of IL‐2 and TGF-β to T cells *via* the use of nanoparticles (NPs) made with polymer poly(lactic‐co‐glycolic acid) (PLGA) could protect lupus mice from systemic autoimmunity by inducing Tregs - but also possibly modulating the activity of additional immune cells (10). Here we show that a key contribution to the suppression of lupus-like disease in BDF1 mice by NPs comes from NK cells and their TGF-β production.

## Methods

### Nanoparticles

Poly(lactic-co-glycolic acid (PLGA) NPs were prepared according to a previously described protocol (10). Briefly, 60 mg PLGA (Durect, Cupertino, CA) were dissolved in 3 ml of chloroform in a glass test tube. Dropwise addition of 200 μl of an aqueous solution containing carrier‐free 1.25 μg IL‐2 with or without 2.5 μg TGF-β (PeproTech, Cranbury, NJ), resulted in a primary emulsion which was sonicated and added dropwise to a continuously vortexed glass test tube containing 4 ml of 4.7% polyvinyl alcohol (PVA) and 0.625 mg/ml avidin–palmitate conjugate. The resulting double emulsion was sonicated in an ice bath before transfer to a beaker containing 200 ml of 0.25% PVA. Particles were allowed to harden by stirring for 3 h at room temperature and then washed 3 times by cycles of pelleting at 18,000*g *and resuspension in Milli‐Q water. The washed NPs were flash‐frozen in liquid nitrogen and lyophilized, to enable long‐term storage at −20°C until use. The NP preparations underwent examination for physical properties, encapsulation metrics, and release kinetics, as previously reported. Size was quantified using dynamic light scattering with a Malvern Zetasizer Nano. NPs were found to have a hydrodynamic diameter of 245.3 ± 2.2 nm with a low polydispersity index, indicating a uniform NP population with a relatively tight size distribution. Cytokine encapsulation and release were checked by BD OptEIA ELISA kits after disrupting the NPs in DMSO and by supernatant analysis. For cell targeting, NPs were diluted in phosphate buffered saline (PBS) and incubated with biotinylated anti-CD2 antibody (clone RM2-5, Thermo Fisher Scientific, Waltham, MA) at a ratio of 5 to 10 μg to 1 mg NPs 10 min before use.

### Mice

(C57BL/6 × DBA/2)F_1_ (B6D2F1/J, hereafter called BDF1) mice and DBA/2 mice were purchased from The Jackson Laboratory (Bar Harbor, ME) and housed in pathogen–free facilities at the University of California, Los Angeles. Lupus-like disease was induced at 8 weeks of age, according to standard protocols, by transferring 1 × 10^8^ DBA/2 splenocytes into BDF1 mice (11). In the recipient mice, the recognition of the host major histocompatibility complex (MHC) antigens leads to lymphoid hyperplasia and elevated production of anti–double‐stranded DNA (anti‐dsDNA) antibodies followed by immune-complex glomerulonephritis that is characterized by a chronic graft versus host disease resembling lupus (11). After the transfer of the DBA/2 splenocytes, individual BDF1 mice were given intraperitoneal (i.p.) injections of vehicle (as control) or 1 mg PLGA NPs loaded with IL‐2 or IL-2/TGF-β and decorated with biotinylated anti‐CD2 antibody, together or not with biotinylated anti‐CD4 antibody (clone GK1.5, Thermo Fisher Scientific). Uncoated NPs with no encapsulated cytokines served as control. The protocol of NPs administration was the following: day 0, day 3, day 6, day 9, day 12 and day 19. In one dose-ranging study, in addition to the group of mice that received NPs at the individual dose of 1 mg each time, a second group received 2 mg and a third group received 4 mg each time. Therefore, the total amount of NPs in the first group was 6 mg, 12 mg in the second group, and 24 mg in the third group. In another set of experiments, mice received i.p. 100 µl of NK-depleting rabbit anti-asialo GM1 (Wako Chemicals, Richmond, VA) or rabbit control serum (Sigma-Aldrich, St. Louis, MO) from day 0, at 4-day intervals, for 2 weeks. Efficacy of NK depletion (>90%) was assessed by flow cytometry using FITC-labeled anti-NK1.1 antibody (clone PK136, Thermo Fisher Scientific).

Mice were monitored at weekly intervals using blood obtained *via* retroorbital bleeding for analyses that included flow cytometry on circulating immune cells, serum creatinine (Abcam, Cambridge, MA) and ELISA measurements of circulating anti-dsDNA antibodies (Alpha Diagnostic Intl., San Antonio, TX). Proteinuria was measured using Albustix strips (Siemens Diagnostics, Irvington, NJ).

In a series of experiments, individual mice received i.p. every other day from day 0, for two weeks, 100 µg anti-TGF-β antibody (clone 1D11.16.8—a neutralizing antibody to all three isoforms of TGF-β that has a circulating half-life of 15.2 h [12]) or the same amount of isotype control antibody (clone P3.6.2.8.1) (both from Novus Biologicals, Centennial, CO). The experiments were approved by the institutional Animal Research Committee.

### Flow Cytometry

Peripheral blood mononuclear cells (PBMCs) or splenocytes were isolated according to standard procedures. Single‐cell suspensions were used for flow cytometry analyses. After red blood cell lysis and Fc blocking, anti‐mouse NK1.1 (FITC-labeled) or H-2K^b^/H-2D^b^ (PE-labeled) (clone 28-8-6, Biolegend, San Diego, CA) or isotype control antibodies were used for staining. Data were acquired on a FACSCalibur™ flow cytometer (BD Biosciences, San Jose, CA) and analyzed using FlowJo™ software (BD, Franklin Lakes, NJ).

### siRNA Transfection and Real-Time PCR

The protocol of siRNA transfection has been described elsewhere (13). Briefly, untouched NK cells isolated using the NK Cell Isolation kit on an autoMACS (Miltenyi Biotec, Auburn, CA) were plated on 12-well plates in complete medium containing 10% fetal bovine serum 24 h before transfection with the Silencer Select siRNA for mouse Tgfb1 (Thermo Fisher Scientific) using the Silencer siRNA Transfection II Kit that also included GAPDH siRNA as positive control and a negative control siRNA with no significant sequence similarity to mouse, rat, or human gene sequences (Silencer siRNA Transfection II Kit). siPORT amine transfection agent was diluted in OptiMEM™ medium (Thermo Fisher Scientific) and used alone as additional control or mixed with 10 nM siRNAs (Tgfb1 or control) before incubation for 30 min. at room temperature. Sorted NK cells were transfected with TGF-β siRNA or negative control complexes and given i.v. to BDF1 mice at a dose of 2.5 × 10^6^ NK cells per mouse the day after induction of lupus-like disease. To control efficiency of siRNA transfection before the adoptive transfer, a small additional aliquot of cells was lysed with TRIzol™ reagent (Thermo Fisher Scientific) for total RNA isolation. 100 ng RNA were used with one-step RT-PCR reagents from Thermo Fisher Scientific using primers and probe combinations described elsewhere (12, 14). For relative quantitation, a standard curve was constructed for each primer and probe set, using total RNA. GAPDH was used as an endogenous control in each experimental set. All samples were run in duplicate.

### Statistical Analyses

Assessment for normal distribution was done by Shapiro-Wilks test. All comparisons among multiple groups were done by one-way ANOVA with Bonferroni’s correction and comparisons between two groups evaluated using (post-hoc) Student’s *t* test, unless indicated otherwise. Data were analyzed using GraphPad Prism software; *P *values of <0.05 were considered significant.

## Results

### NK Cells Contribute to the Protection of BDF1 Mice From Lupus-Like Disease Conferred by IL-2/TGF-β-Loaded Nanoparticles

We recently reported that NPs containing IL-2 and TGF-β targeted to T cells induced large numbers of CD4^+^ and CD8^+^ Tregs *in vivo* that protected BDF1 mice from lupus-like disease (10). In those studies, cytokine-laden NPs were coated with anti-CD4 and anti-CD2 antibodies (the latter being responsible for the induction of CD8^+^ Tregs). To further those findings, we asked whether NK cells could as well be targets of the NPs. This is because NK cells express high levels of CD2 molecules on their cell surface (15), have immunomodulatory properties (in addition to their cytotoxic properties) (16), and anti-CD2 antibodies stimulate NK cells that inhibit B-cell production of antibodies *via* TGF-β (15).

**Figure 1:**
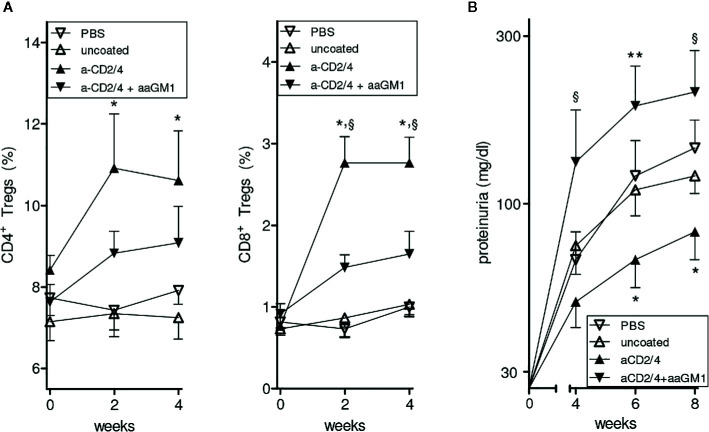
Depletion of NK cells reduces the expansion of CD4^+^ and CD8^+^ Tregs induced by NPs loaded with IL-2 and TGF-β and decorated with anti-CD2/CD4 antibodies. Symbols represent the different treatments (n = 6 mice per group); error bars show the mean ± SEM. **(A)** Percentages of peripheral CD4^+^ (left) and CD8^+^ (right) Tregs at the indicated time points after treatment. *P<0.05 in the comparison between anti-CD2/4 Ab-coated NPs vs. uncoated NPs; ^§^ *P*<0.04 between anti-CD2/4 NP-treated mice depleted (anti-asialo GM1, aaGM1) of NK cells vs. non NK-depleted mice. **(B)** Proteinuria at the time points indicated for the mice in A. *P<0.05 between anti-CD2/4 Ab-coated NPs vs. uncoated NPs; ^§^ *P*<0.05 and **P<0.005 in the comparison between mice treated with anti-CD2/4 NP-treated mice depleted (aaGM1) of NK cells vs. non NK-depleted mice.

To investigate possible contributions of NK cells to the protective effects conferred by the NPs in BDF1 mice, we repeated the experiment of prevention of lupus-like disease induced by IL-2/TGF-β-loaded NPs (10) but this time we depleted (or not, as control) the circulating NK cells in the NP-treated animals (see *Methods*). As shown in **Figure 1A**, NK cell depletion markedly reduced the number of circulating CD4^+^ and CD8^+^ Tregs and exacerbated renal disease in NP-treated mice (**Figure 1B**). These results indicate that NK cells modulate the tolerogenic activity of the NPs in BDF1 mice.

### NK Cells That Expand in Nanoparticle-Treated BDF1 Lupus Mice Are Host-Derived

**Figure 2:**
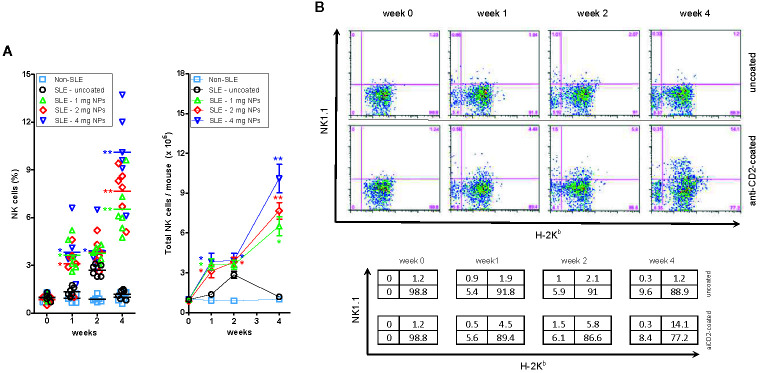
Host NK cells expand numerically in BDF1 with lupus-like disease after treatment with CD2-targeted NPs loaded with IL-2 and TGF-β. **(A)** Left: Percentage numbers of circulating NK cells in PBMCs from individual untreated BDF1 mice (“non-SLE”, i.e. not receiving DBA/2 splenocytes and NPs) and in lupus BDF1 mice treated with different doses of NPs encapsulating IL-2 and TGF-β: 1, 2, 4 mg each time. Uncoated NPs at the intermediate dose (2 mg) served as control. See *Methods* for details. Right: Total numbers of NK cells (mean ± SE) in mice under the conditions as in the left panel. *P* = *<0.05, **0.005 in the comparisons with lupus BDF1 mice treated with uncoated NPs. **(B)** NK cells that expand in BDF1 lupus mice after treatment with NPs are host-derived (H-2K^b+^). After treatment with 4 mg anti-CD2 Ab-coated or control (uncoated) NPs according to the protocol described in the *Methods*, monitoring by flow cytometry used H-2 markers to discriminate NK cells (NK1.1^+^) from DBA/2 donors (H-2K^b−^) vs. those from BDF1 recipients (H-2K^b+^). Percentages in the quadrants are shown in the table.

A dose-dependent increase in the frequency of circulating NK cells in BDF1 lupus mice that had received CD2-targeted NPs loaded with IL-2/TGF-β was not seen in non-lupus mice or in mice that had received uncoated NPs (**Figure 2A**).

To understand whether the expanded NK cell population derived from the host (BDF1 mice) or from the donor (DBA/2 mice), we evaluated by flow cytometry the surface expression of H-2 molecules on the expanded NK cells. The parental haplotypes of the recipient BDF1 lupus mice are H-2^b^ (C57BL/6) and H-2^d^ (DBA/2), so the transferred H-2^d^ splenocytes from DBA/2 mice do not stain with anti-H-2^b^ antibodies. Therefore, H-2^b^ NK cells must only be of host origin. Flow cytometry analyses showed that NP administration increased the number of H-2^b^ (host) NK cells at two weeks, expanding further at four weeks (**Figure 2B**). There was neither an increase in circulating H-2^b^ cells in BDF1 mice that did not receive NPs nor an increase in H-2^d^ donor NK cells (**Figure 2B**), indicating that the expansion of NK cells in NP-treated BDF1 lupus mice was the result of an increase in the (relative and absolute) numbers of host-derived NK cells.

### NP-Mediated Expansion of NK Cells Associates With the Suppression of Anti-dsDNA Antibodies and Reduced Lupus Disease Manifestations in BDF1 Mice

**Figure 3:**
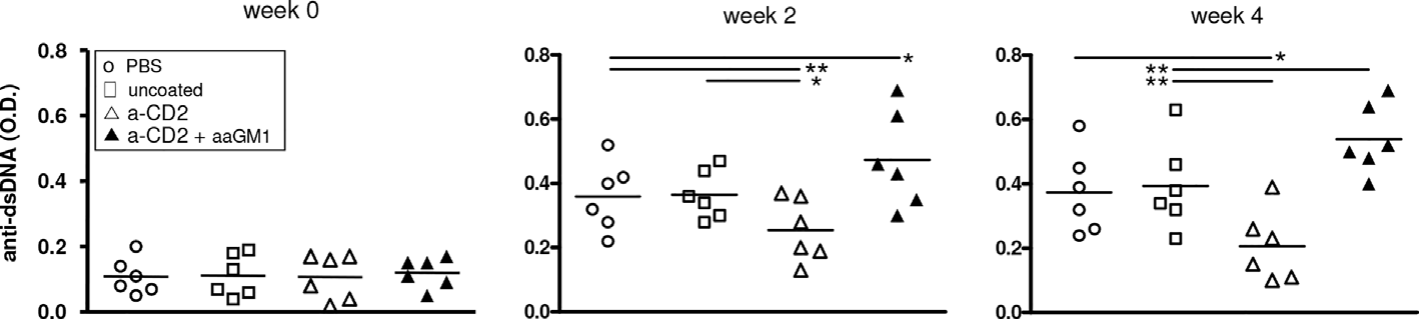
Depletion of NK cells in BDF1 lupus mice that received NK cell-targeted NPs (anti-CD2 Ab loaded with IL-2) associates with increased levels of serum anti-dsDNA autoantibodies. Conversely, treatment with CD2 Ab (NK cell)-targeted NPs associates with suppression of anti-DNA autoantibodies. NK cells were depleted by administering anti-asialo GM1 (aaGM1) as indicated in the *Methods*. Monitoring of individual mice and group means are reported at week 2 and week 4 post-induction of SLE (time 0); *P<0.05, **P<0.01.

**Figure 4:**
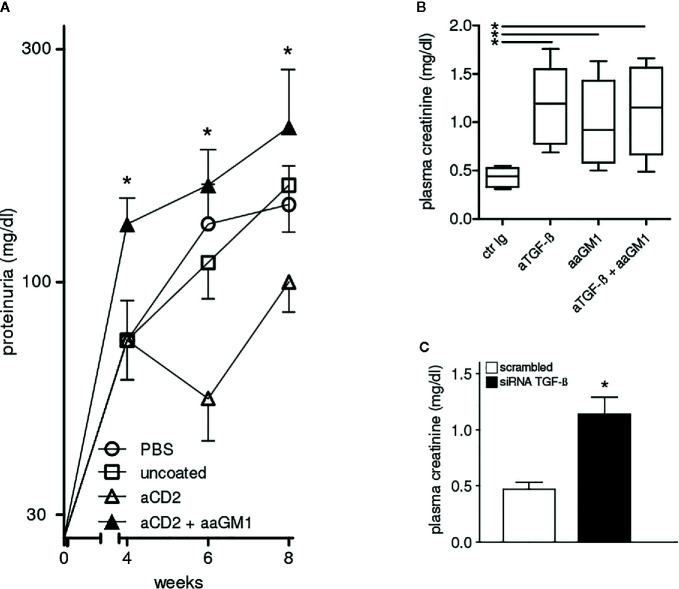
Protection from lupus nephritis of BDF1 mice treated with CD2 (NK cell)-targeted NPs depends on NK cells and TGF-β. **(A)** NK cell depletion with anti-asialo GM1 (aaGM1) accelerates proteinuria in BDF1 lupus mice (see *Methods* for details). Mice (n=6 per group) were monitored for 8 weeks post-induction of SLE. Data show the mean ± SE; *P<0.01 in the comparison between anti-CD2 Ab-targeted NPs mice that had been depleted or not of NK cells. **(B)** Administration of anti-TGF-β antibodies (aTGF-β) to BDF1 lupus mice (n = 6 per group) abrogates the NK cell-mediated protective effects associated with the treatment with CD2 Ab-targeted NPs. Serum creatinine was measured 3 weeks after treatment with anti-TGF-β Ab or control Ab (ctr Ig). *P<0.04 in the comparison with ctr Ig. **(C)** 2.5 × 10^6^ NK cells pretreated with TGF-β siRNA or control siRNA were transferred into individual BDF1 lupus mice (n = 3 per group) 1 day after lupus disease induction. Serum creatinine was measured after 3 weeks. *P<0.01 by the Student’s *t* test.

Because of the central role of autoantibodies in lupus pathogenesis and the finding that NK cells can suppress B-cell production of antibodies *in vitro* and *in vivo* (17–19), we investigated whether the NK cells influenced autoantibody levels in BDF1 lupus mice. Moreover, since anti-CD2 antibodies induce NK cells to produce TGF-β (15) we considered the possibility that the production of this cytokine by NK cells could substitute for that encapsulated in the NPs. In experiments with NPs that contained only IL-2, NK cells markedly influenced the serum levels of autoantibodies in BDF1 lupus mice that received NPs, as depletion of NK cells with anti-asialo GM1 resulted in significantly increased titers of anti-dsDNA autoantibodies (**Figure 3**) and accelerated renal disease manifestations (proteinuria) (**Figure 4A**).

### Beneficial Effects of NK Cells and TGF-β on the Disease Manifestations in BDF1 Lupus Mice

Although NK cells can produce large amounts of interferon γ (IFN-γ), these cells can also be major producers of TGF-β (15). Considering that TGF-β contributes to the suppression of the autoimmune response in BDF1 lupus mice (3, 4), we studied the effects of TGF-β inhibition in BDF1 mice. The readout in these experiments was the measurement of serum creatinine. Increased serum creatinine levels are an early indicator of kidney injury and reflect a progression to renal insufficiency and end-stage renal disease in lupus nephritis (20).

The comparison between BDF1 lupus mice that received NK cell-targeted NPs together with anti-TGF-β antibody vs. mice that received an irrelevant control antibody indicated that the inhibition of TGF-β associated with a significant increase in serum creatinine (**Figure 4B**). The contributing role of NK cells was confirmed by the finding of elevated serum creatinine in BDF1 lupus mice that had been depleted of NK cells with anti-asialo GM1 (**Figure 4B**).

The finding that the combination of anti-asialo GM1 and anti-TGF-β antibodies did not influence serum creatinine levels further suggested the possibility of a common mechanism (**Figure 4B**). To test this possibility, we sorted NK cells from BDF1 mice treated with NK cell-targeted NPs and inhibited their production of TGF-β using siRNA technology. Transfection of NK cells with negative control siRNA served as control. The adoptive transfer of 2.5 × 10^6^ TGF-β−sufficient NK cells into BDF1 lupus mice at the time of disease induction did not affect serum creatinine levels that were instead increased in BDF1 mice receiving an identical number of NK cells but TGF-β−deficient (TGF-β siRNA) (**Figure 4C**). Together, these results indicate that the NK cell-mediated protective effects on kidney disease in BDF1 mice are TGF-β-dependent.

## Discussion

We report that CD2-targeted NPs loaded with IL-2 can induce an expansion of NK cells that suppress lupus-like disease in BDF1 mice through TGF-β-dependent mechanisms. These results advance and extend our past findings that had shown a therapeutic potential of NP-based immunotherapy in mice with lupus-like disease.

Several strategies have been developed to suppress the production of pathogenic autoantibodies in systemic autoimmune diseases. In SLE, the use of tolerogenic approaches has generally focused on the induction of immunoregulatory adaptive immune cells, either through *ex vivo* conditioning of immune cells or through the expansion *in vivo* of the pool of circulating Tregs (6). We recently expanded Tregs *in vivo* in lupus mice using NPs that delivered tolerogenic cytokines to T cells that expressed CD4 and CD2 (10). In those studies, we unexpectedly observed an involvement of additional immune cells that contributed to the protection from disease. Specifically, NPs better protected lupus mice when concomitantly targeted to CD4^+^ and CD2^+^ cells, being the suppressive activity on autoantibody production and disease manifestations superior to the sum of the activities deriving from CD4^+^ and CD8^+^ Tregs (10). Since NK cells express CD2 on their cell surface, we investigated a possible involvement of NK cells because CD2 acts synergistically with CD16 for NK cell activation and this molecule is critical in the control of the antibody response (21) modulated by NK cells at the T and B cell levels (17–19, 22). NK cells are also known to produce cytokines, including IFN-γ, TNF-α, GM-CSF (23), and are the principal lymphocyte source of TGF-β (being both the inactive precursor of TGF-β and active TGF-β produced spontaneously by NK cells) (24). After anti-CD2/anti-CD16 antibody stimulation, NK cells produce large amount of TGF-β and IL-10 (25), and anti-CD2 antibodies alone increase TGF-β production and promote NK cell-mediated suppression of autoantibody production (26). Our findings combine those observations by showing a CD2-mediated induction of NK cells that mediate suppressive activities through TGF-β-dependent mechanisms.

Although the reduction in titers of anti-dsDNA autoantibodies in mice receiving CD2-targeted NPs at weeks 2 and 4 (**Figure 3**) appeared as insufficient to suppress the initial renal damage that develops in lupus BDF1 mice, it resulted in (more evident) beneficial effects after a prolonged time frame (**Figure 4A**).

CD2 is an adhesion molecule that also operates as a costimulatory molecule (27). Most studies on CD2 in autoimmunity have focused on the expression of this molecule on T cells. For example, the acute, benign immune suppression induced by anti-CD2 antibodies in multiple sclerosis has been investigated with a focus on the patients’ T cells (28). Also, the immunosuppressive activity of the CD2‐specific fusion protein alefacept in patients with autoimmune diabetes has been linked to a depletion of CD4^+^ and CD8^+^ central memory T cells (Tcm) and effector memory T cells (Tem) but specific studies on NK cells have been missing (29, 30). In view of our results, it might be interesting to investigate how much of the immunosuppressive effects of anti-CD2 antibodies on autoimmune responses involve NK cells and whether a synergy exists between anti‐CD2 antibodies and multivalency (whereby the same antibodies might sequentially or concomitantly operate on both NK cells and T cells). It should also be interesting to know whether the effects of anti‐CD2 antibodies might be amplified by the local acidic microenvironment created by the NPs – which might favor the conversion of endogenous latent TGF-β to its active form (i.e., potentiating its activity).

These non-mutually exclusive possibilities could be the focus of future investigations that should also include the evaluation of the role of basophils in the system. Indeed, anti-asialo GM1 depletes basophils, in addition to NK cells (31). We found that the treatment of BDF1 mice with anti-asialo GM1 did concomitantly deplete NK cells and basophils *in vivo* (data not shown), suggesting that the role of basophils might be a possible contributing factor to the results, although this aspect requires direct investigations.

Notwithstanding these considerations, our study identifies for the first time a protective role of NP-induced NK cells in SLE, envisioning a possibility of NP-driven, cell-targeted immunotherapy in the disease.

## Data Availability Statement

The raw data supporting the conclusions of this article will be made available by the authors, without undue reservation.

## Ethics Statement

The animal study was reviewed and approved by Animal Research Committee of UCLA.

## Author Contributions

ALC and DH designed the study. AL, SB, and ALC performed the experiments. DH, AL, SB, GC, GM, TF, and ALC analyzed and discussed the results and gave critical intellectual contributions. ALC wrote the manuscript. DH contributed to its final editing. All authors contributed to the article and approved the submitted version.

## Funding

Supported in by part by the NIH grants HD97531 and AI154935 to ALC. GM is funded by grants from Fondazione Italiana Sclerosi Multipla (FISM 2016/R/18 and 2018/S/5) and Progetti di Rilevante Interesse Nazionale (PRIN) 2017 K55HLC 001. 

## Conflict of Interest

DH is an officer of General Nanotherapeutics LLC and owns stock. DH and TF own stocks and stock options at Toralgen, Inc.

The remaining authors declare that the research was conducted in the absence of any commercial or financial relationships that could be construed as a potential conflict of interest.
